# Pr^3+^ Ion-Substituted Ni-Co Nano-Spinel Ferrites: Their Synthesis, Characterization, and Biocompatibility for Colorectal Cancer and Candidaemia

**DOI:** 10.3390/ph16101494

**Published:** 2023-10-20

**Authors:** Suriya Rehman, Balasamy Rabindran Jermy, Irfan A. Rather, Jamal S. M. Sabir, Suhailah S. Aljameel, Munirah A. Almessiere, Yassine Slimani, Firdos A. Khan, Abdulhadi Baykal

**Affiliations:** 1 Department of Epidemic Diseases Research, Institute for Research & Medical Consultations (IRMC), Imam Abdulrahman Bin Faisal University, Dammam 31441, Saudi Arabia; 2 Department of Nanomedicine Research, Institute for Research & Medical Consultations (IRMC), Imam Abdulrahman Bin Faisal University, Dammam 31441, Saudi Arabia; rjermy@iau.edu.sa; 3 Department of Biological Sciences, Faculty of Science, King Abdulaziz University, Jeddah 21589, Saudi Arabia; ammm@kau.edu.sa; 4 Center of Excellence in Bionanoscience Research, King Abdulaziz University, Jeddah 21589, Saudi Arabia; 5 Department of Chemistry, College of Science, Imam Abdulrahman Bin Faisal University, Dammam 31441, Saudi Arabia; ssaljameel@iau.edu.sa; 6 Department of Biophysics Research, Institute for Research & Medical Consultations (IRMC), Imam Abdulrahman Bin Faisal University, Dammam 31441, Saudi Arabia; malmessiere@iau.edu.sa (M.A.A.); yaslimani@iau.edu.sa (Y.S.); 7 Department of Stem Cell Research, Institute for Research & Medical Consultations (IRMC), Imam Abdulrahman Bin Faisal University, Dammam 31441, Saudi Arabia; fakhan@iau.edu.sa; 8 Food Engineering Department, Faculty of Engineering, Istanbul Aydin University, Florya, Istanbul 34295, Turkey; abaykal@iau.edu.sa

**Keywords:** spinel ferrites, Pr substitution, biomedical nanotechnology, disease, sonochemical synthesis

## Abstract

Nanotherapeutics have attracted tremendous research interest in the modern pharmaceutical and biomedical industries due to their potential for drug development, targeted delivery, and therapeutic applications. Therefore, the current study underpins the synthesis of praseodymium ion (Pr^3+^)-substituted Ni_0.5_Co_0.5_Fe_2_O_4_ nano-spinel ferrites, (Co_0.5_Ni_0.5_Pr_x_Fe_2−x_O_4_ (0.0 ≤ x ≤ 0.10) NSFs, CoNiPr (x ≤ 0.10) NSFs) via the sonochemical route for its application as a nanotherapeutic treatment option. The synthesized nanomaterial was characterized using various analytical techniques, including scanning/transmission electron microscopy (SEM) and X-ray powder diffractometry (XRD). After substitution with Pr (x = 0.08), the particle size, polydispersity index, and zeta potential analysis indicated an increase in hydrodynamic diameter, with an average zeta potential value of −10.2 mV. The investigation of CoNiPr (x ≤ 0.10) NSFs on colorectal cancer (HCT-116) cells demonstrated a significant effect on cancer cell viability. The inhibitory concentration (IC_50_) of CoNiPr (x ≤ 0.10) NSFs was between 46 ± 0.91 and 288 ± 8.21 for HCT-116 cells. The effect of CoNiPr (x ≤ 0.10) NSFs on normal human embryonic kidney (HEK-293) cells showed a reduction in the HEK-293 cell viability; however, the cell viability was better than HCT-116. The NSFs treatment also showed morphological changes in cancer cell nuclei, as revealed by DAPI (4′,6-diamidino-2-phenylindole), nuclear disintegration, and chromatic fragmentation, which are signs of apoptosis or programmed cell death. To examine the potential antifungal effects of CoNiPr NSFs on *Candida albicans*, known to cause candidemia among cancer patients, the viability of the cells was assessed post treatment with CoNiPr (x ≤ 0.10) NSFs. The increasing ratio of dopant had a moderate impact on the percentage of cell viability loss of 42, 44, and 43% with x = 0.06, 0.08, and 0.10, respectively. These results reinforce that increased dopant significantly impacts the antifungal properties of the synthesized nanomaterial. These findings support the idea that NSFs might be useful in pharmaceuticals.

## 1. Introduction

Antimicrobial resistance poses a serious public health risk globally [1]. The abuse of antimicrobials for treating infections is the main reason for this situation, especially in agriculture, veterinary medicine, and industry [2]. Increased antimicrobial utilization has resulted in gene mutations and drug resistance among pathogens [3]. This existing health risk has indicated an urgent for developing new antimicrobial drugs to control and treat life-threatening infections. On the other hand, cancer constitutes the primary cause of death worldwide, for which substantial means of therapies need to be offered [4]. Therefore, nanotechnology has emerged as a novel field to combat antimicrobial resistance and improved anticancer therapy. Among nanomaterials, metal and metal oxide NPs and spinel ferrites (SFs) have attracted colossal attention, as numerous studies have reported their enhanced action toward resistant pathogens and their anticancer potential [5,6,7].

There has been growing interest in using nanomaterials for biomedical applications in recent years due to their unique physicochemical properties, which allow for targeted drug delivery and enhanced efficacy. Nanomaterials are considered as one potential candidates for effective drug delivery systems due to their efficient characteristics to penetrate the biological barriers, reduced he drug dose to be administered and minimum side effects [8,9].

SFNs are the recent advancement in the field of magnetic nanoparticles. They carry both the characteristics of magnetic and useful electric properties. SFNs have emerged as a promising class of agents for anticancer therapy due to their biocompatibility, high magnetic properties, and ability to generate heat when exposed to an alternating magnetic field. Studies have shown that SFNs can effectively kill cancer cells in vitro and in vivo by inducing hyperthermia and reactive oxygen species (ROS) generation, leading to the apoptosis and necrosis of the cancer cells [10,11]. Highly efficient T2 CoFe_2_O_4_ cobalt nanoparticles vectorized for internalization in cancer cells were studied by Mazario et al. [12]. Fiaz et al. used a green synthesis route to synthesize cobalt ferrite nanoparticles for anticancer, antidiabetic, and antibacterial studies [13]. Barani et al. investigated the theranostic application of cobalt ferrite nanoparticles [14]. Jermy et al. studied the tuning of pH-sensitive chitosan and cisplatin over spinel ferrite/silica nanocomposite for anticancer studies using an MCF-7 cell line [15]. Poorhossein et al. designed a multifunctional nanoplatform based on PEGylated cobalt ferrite magnetic nanoparticles containing capecitabine for cancer theranostics. In addition, SFNs have attracted significant attention due to its rare features and potential applications, such as catalysts, magnetic bulk cores, magnetic fluids, microwave absorbers, diagnostics, pharmaceutics, etc [16]. Due to the larger ionic radii, rare earth metals mainly produce ferrites with partially substituted Fe^3+^ ions, resulting in structural and characteristic modifications [17]. The doping concentration of RE ions in spinel ferrites can modify their magnetic characteristics by directly affecting the magnetic anisotropy constant [18]. The effect of rare earth Nd3þ doping content on the physical, structural, and magnetic properties of CoeNi spinel ferrite nanoparticles was also studied [7]. The underlying mechanism of such nanomaterial interaction with biological systems mostly depends on the pore formation of the cellular membranes. The electrical characteristics of such nanoparticles affect the cancer cells differently than the normal healthy cells. Cancer cells show unique bioelectrical nature where electrophysiological examination of various tumor cells exhibited a depolarization. This depolarization of membrane leads to potential susceptibility of cancer cells to electroporation. This mechanism potentially allows delivery within the cells via induced pores. By adding RE ions into the spinel lattice, the interactions seem to change the electrical, magnetic, and biological impact in the ferrites. Also, different RE metals are known to impact the ferrites differently [19]. Several studies have reported that the substitution of Fe^3+^ ions with Pr^3+^ ions affects the structural, magnetic, and electrical properties of various spinel ferrites. It was demonstrated that these samples could be potential candidates for medical applications like magnetic resonance imaging [20,21]. In the current study, we synthesized and investigated the influence of Pr^3+^ ions with NiCo nano-spinel ferrites on biological activities. The ultrasonic approach was used to synthesize the nanoparticles. The structural, anticancer, and antifungal properties were studied to confirm the effect of Pr^3+^ ions on the properties of Ni_0.5_Co_0.5_Fe_2_O_4_ NSFs.

Therefore, this study aims to synthesize Ni-Co nano-spinel ferrites doped with Pr^3+^ ions and investigate their potential as a therapeutic agent for colorectal cancer and candidaemia.

## 2. Results and Discussion

### 2.1. Microstructure and Morphology

**Figure 1:**
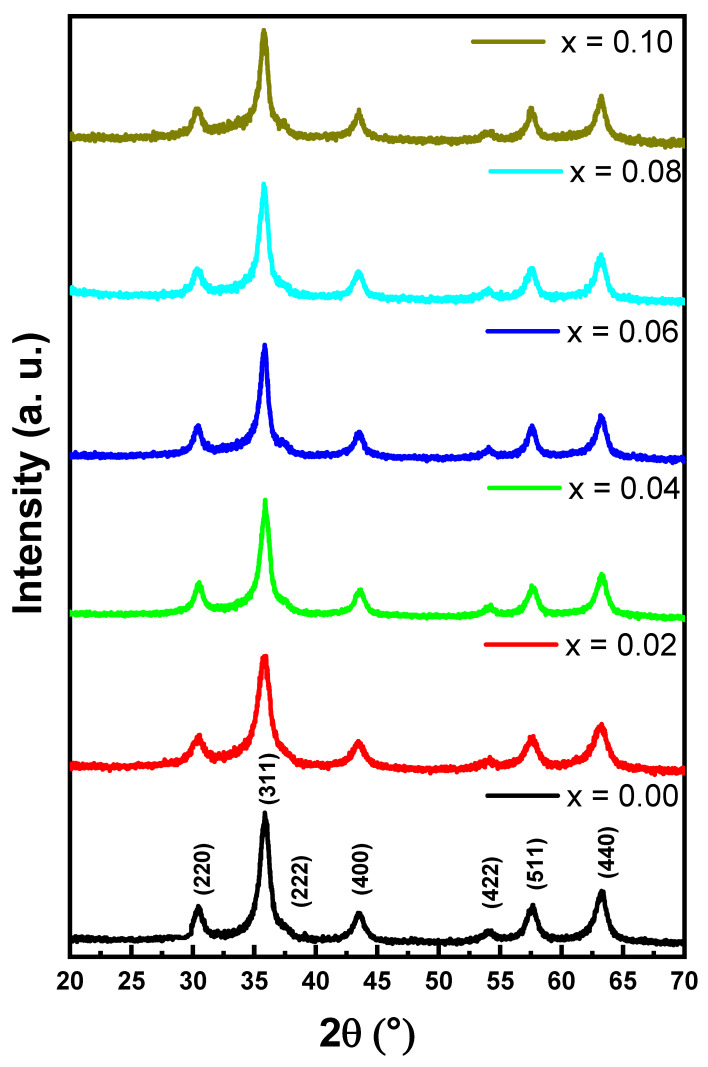
XRD powder patterns of CoNiPr (0.00≤x≤0.10) NSFs.

The phase of CoNiPr (x ≤ 0.10) NSFs was analyzed via XRD powder patterns, as illustrated in Figure 1. The samples exhibited good agreement with the cubic spinel ferrite phase (JCPDS Card No. 15-0806). The cell parameters and crystal size were estimated using Match 3! Software (Crystal Impact, version 3.15 Build 278, Karlsruhe, Germany). It was found that the cell parameter lattice constant (a) was in the range of 8.335 (0) to 8.326 (9) Å, while the crystal size was between 9.5 and 11.3 nm. 

**Figure 2:**
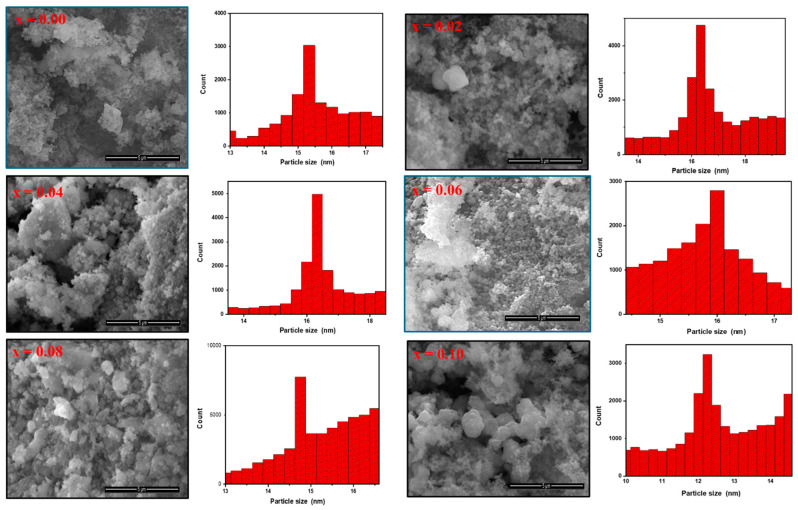
SEM surface analysis and grain size histograms of CoNiPr (0.00 ≤ x ≤ 0.10) NSFs.

**Figure 3:**
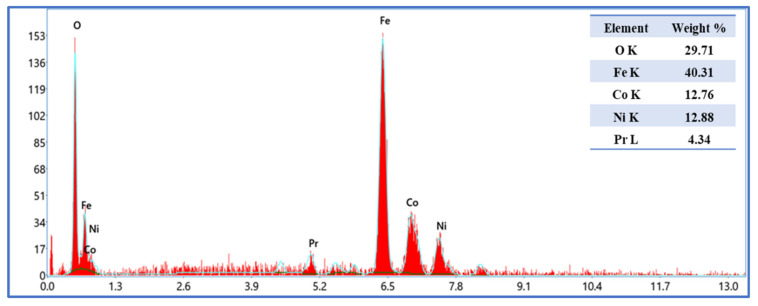
EDX spectrum showing the elemental analysis of CoNiPr (x = 0.04) NSFs.

Surface analysis of CoNiPr (x = 0.00, 0.02, 0.06, and 0.10) NSFs was performed through SEM, as seen in Figure 2. SEM showed that the nanoparticles were obtained with some aggregation behavior. The agglomeration of magnetic particles is due to the high surface energy between the prepared magnetic nanoparticles and magnetic dipole–dipole interactions. Magnetic dipole forces may also contribute to the observed interparticle forces. The strength of these interparticle bonds determines their physical properties and applicability to material synthesis. The images unveiled the clustering of small spherical particles caused by the magnetic nature of the materials. Additionally, there was a slight change in particle size with increasing Pr ratios due to the hindrance in particle growth doped by rare earth caused by the difference of ionic radii between the doped and the host lattice elements [21,22]. The particle size distribution was calculated through Image J software version 1.53t, (New York, United States )and it was found in the range of 10 to 17 nm. The elemental analysis of CoNiPr (x = 0.04) NSFs was confirmed via EDX, as seen in Figure 3. The spectra disclosed the presence of Ni, Fe, Co, Pr, and O, which approved the efficiency of the synthesis method without the presence of any impurities. 

### 2.2. Nitrogen Physisorption

**Figure 4:**
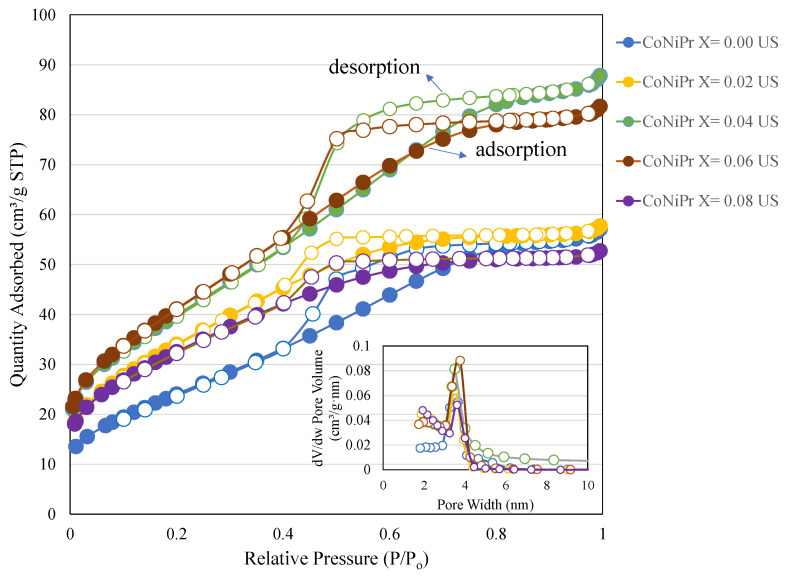
N_2_ adsorption–desorption and pore size distribution curves of CoNiPr (x = 0.00, x = 0.08) NSFs.

The surface characteristics of CoNiPr (x ≤ 0.10) NSFs synthesized using the sonochemical route were analyzed using the nitrogen adsorption and desorption technique (Figure 4). Table 1 shows the surface area, pore volume, and pore size characteristics of CoNiPr x = 0.00, 0.02, 0.04, 0.06, and 0.08, respectively. CoNiPr (x ≤ 0.10) NSFs without Pr (x = 0.00) exhibit a typical H2 hysteresis loop corresponding to that of cubic-shaped pores [23]. For CoNiPr (x ≤ 0.10) NSFs with x = 0.02, 0.04, 0.06, and 0.08, variation in the pore width reflects the influence of Pr on narrow pore body distributions with a wide neck. In the case of CoNiPr (x = 0.02) NSFs, adding Pr significantly reduces the capillary condensation width, indicating a disordered mesopore formation interconnecting the narrow necks. The surface area increases from 91 m^2^/g to 125 m^2^/g. A further increase in the addition of Pr (x = 0.04 and 0.06) and an increase in the vertical update of nitrogen reflect the mesopore generations due to the structured cubic pores. The surface area increases to a maximum of 151 m^2^/g with a pore volume of 0.12 cm^3^/g. The average pore size distribution ranged between 2.8 and 3.3 nm. Further, an increase in Pr (x = 0.08) reverts the surface area to 117 m^2^/g with a pore volume of 0.08 cm^3^/g. In line with this, the pore diameter also slightly reduces to 2.8 nm.

The zeta potential measurements of CoNiPr x = 0.00 and 0.08 were measured to study the colloidal state stability of nanoparticles (Table 2). The effective diameter of CoNiPr x = 0.00 was 1327 nm. After substitution with Pr (x = 0.08), the particle diameter increases to about 1923 nm. The surface charge of CoNiPr x = 0.00 was −6.13 mV, which changed to −10.2 mV with Pr substitution.

This study shows that the substitution of larger rare earth Pr^3+^ metal ions into Ni-Co nano-spinel ferrites (by increasing the Pr ratios) induces textural variations. The textural analysis using the nitrogen adsorption technique shows that increasing the Pr substitution (x = 0.00–0.06) improves the surface characteristics, while a further increase in the Pr ratio affects the substitution of Fe^3+^ ions leading to reduced surface area, pore volume, and pore size distribution. 

### 2.3. Antifungal Activity

**Figure 5:**
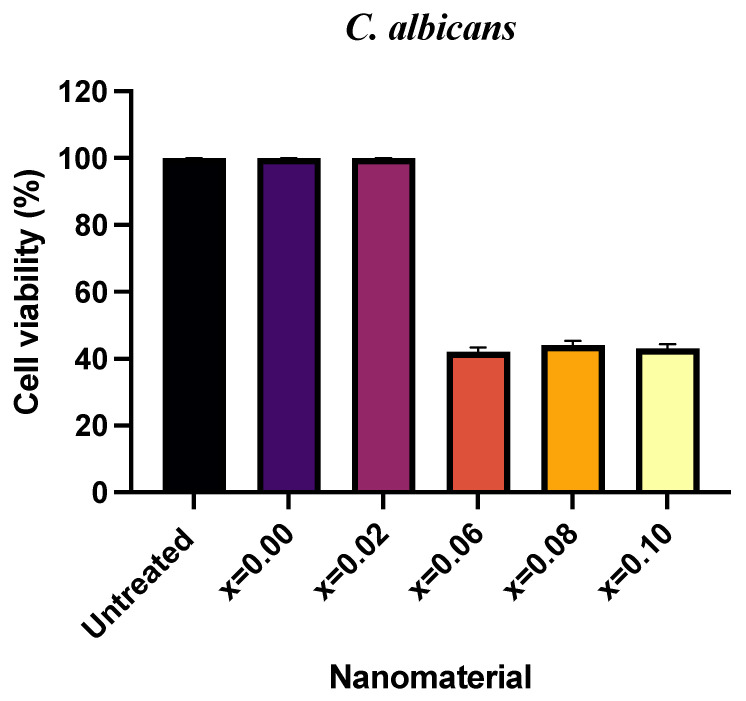
Graph showing the cell survival percentage after treatment with CoNiPr (x ≤ 0.10) NSFs at 16 mg/mL.

**Figure 6:**
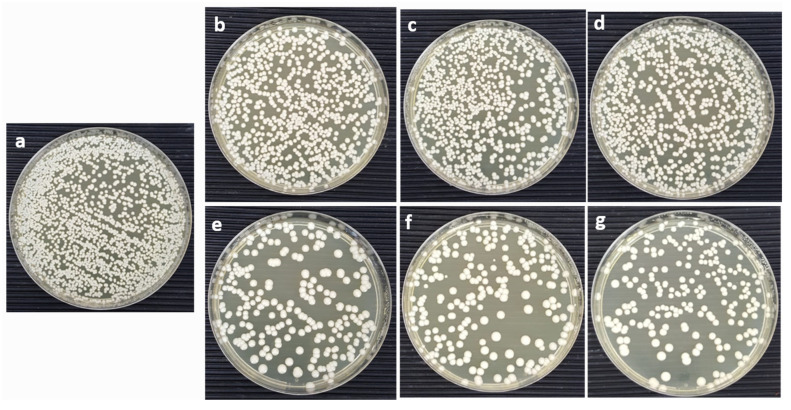
Culture Agar plates representing the CFU after treatment with CoNiPr (x ≤ 0.10) NSFs. (**a**) Untreated, (**b**) x = 0.0, (**c**) x = 0.02, (**d**) x = 0.04, (**e**) x = 0.06, (**f**) x = 0.08, and (**g**) x = 0.1 at 16 mg/mL.

The antifungal action of CoNiPr (x ≤ 0.10) NSFs against *C. albicans* was measured by obtaining cell survival after treatment using the CFU method. The culture was treated with 16 mg/mL of each ratio, and an untreated culture was taken as the control. The increasing ratio of dopant has a moderate impact on the percentage of cell survivability, as presented in Figure 5. It was seen that x = 0.00 and 0.02 showed 100% cell viability similar to the untreated culture, while a significant loss in cell survival of 42, 44, and 43% was seen with x = 0.06, 0.08, and 0.10, respectively. It is evident from the CFU plates that the cell count was marginally seen to be reduced with x = 0.06, 0.08, and 0.10. In contrast, the cell number of plates was found to be similar to the untreated plate and was therefore unaffected by the treatment with x = 0.0, 0.02, and 0.04, respectively (Figure 6). 

### 2.4. Morphogenesis Study Using SEM

**Figure 7:**
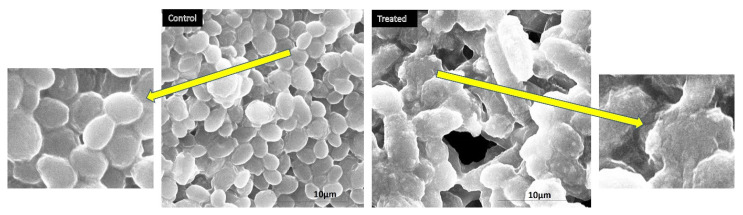
Representative SEM micrographs indicating the structural changes in *C. albicans* brought about by the treatment of CoNiPr (x = 0.08) NSFs and untreated cells (control).

The morphogenesis study indicating the impact of CoNiPr (x ≤ 0.08) NSFs) on Candida was conducted by using SEM. A representative picture has been presented for morphogenesis studies. It can be seen in Figure 7 that the treated cells appear to be distorted, showing the loss of cell integrity. This could be due to the interaction of the nanomaterial with the cellular membranes leading to ROS generation, thereby causing the cell to disrupt and, finally, cell death. On the other hand, the untreated *Candida* cells appear to be normal in shape, size, and structure.

The obtained results are in agreement with a number of studies published previously [24,25]. However, the exact mechanism of the effectiveness of spinel ferrite nanoparticles for bioactivity is still not completely unveiled. However, a few studies have reported that the bioaction of spinel NPs has been largely directed toward the adsorption and penetration of cells, resulting in the disruption of the cellular membrane [26,27,28]. In the current study, the cellular images depict the NP attachment to the cell surface and make the damage of the membrane visible, which has also been reported in several other studies. Our results clearly indicate the possibility of ROS generation, resulting in electrostatic interactions that cause damage to the cells. Studies by Sanpo on spinel-metal-substituted cobalt ferrite reported the potential of such NPs to penetrate cellular surfaces [29]. However, not many studies have been conducted on fungal cells; therefore, our current results indicate the need to unravel the interaction mechanisms and the following cascades between spinel ferrites and fungal cells.

### 2.5. Effect of CoNiPr (x ≤ 0.10) NSFs on Cancer Cell Viability 

**Figure 8:**
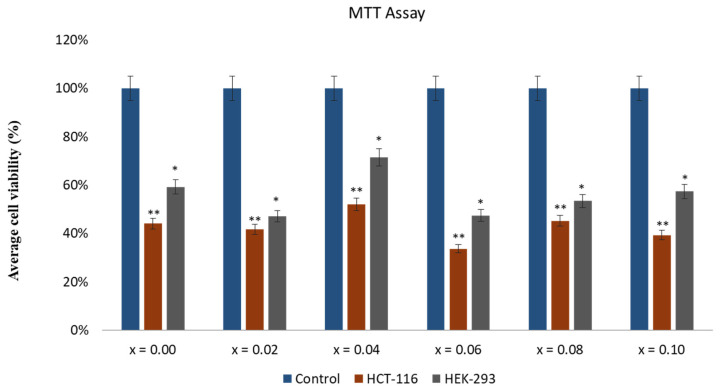
Graph representing the impact of CoNiPr (x ≤ 0.10) NSF treatment on HCT-16 cell viability via MTT assay after 48 h; * *p* < 0.05; ** *p* < 0.001.

The effect of CoNiPr (x ≤ 0.10) NSFs on HCT-116 cells was investigated, and based on MTT assay, there was a significant reduction in HCT-116 cells after the treatment of CoNiPr (x ≤ 0.10) NSFs. The inhibitory concentration (IC_50_) of CoNiPr (x ≤ 0.10) NSFs was calculated, and it was in the range of 46 ± 0.91 to 288 ± 8.21 for HCT-116 cells (Table 3). The effect of CoNiPr (x ≤ 0.10) NSFs was also examined on the normal HEK-293 cells; there was a decrease in HEK-293 cell viability. Nevertheless, the cell viability was greater than that observed in HCT-116 cells (Figure 8), which suggests that CoNiPr (x ≤ 0.10) NSFs are more toxic to cancer cells than normal cells. This is the first study on the anticancer activity of CoNiPr (x ≤ 0.10) NSFs against HCT-116 cells. Few studies have also shown that the treatment of various nanoparticles showed anticancer activities on different cancer cells [27,30]. 

### 2.6. Disintegration of Cancer Cell DNA

**Figure 9:**
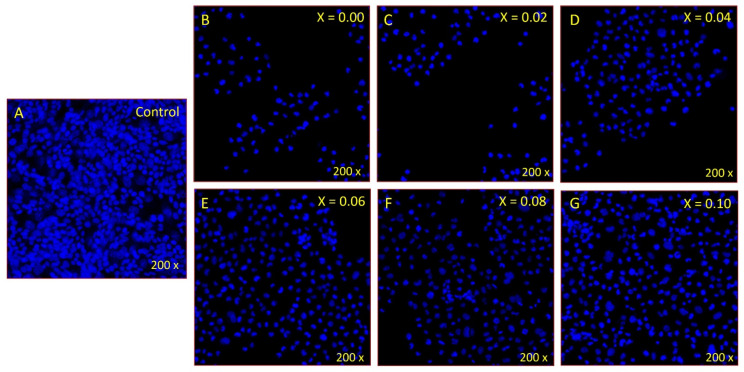
Effect of CoNiPr (x ≤ 0.10) NSFs on HCT-116 cancer cells post 48 h of treatment using DAPI staining. The images clearly depict the effectiveness of nanoparticle treatment at the concentration of (IC_50_) compared to untreated cells. (**A**) Untreated, (**B**) x = 0.0, (**C**) x = 0.02, (**D**) x = 0.04, (**E**) x = 0.06, (**F**) x = 0.08, and (**G**) x = 0.1.

DAPI (4′,6-diamidino-2-phenylindole) staining was performed to evaluate the effect of NSFs on cancer cell nuclei, as DAPI strongly binds to A-T-rich regions in cancer cell DNA. DAPI is a blue fluorescent DNA stain that is usually used to detect apoptosis. CoNiPr (x ≤ 0.10) NSF treatment substantially reduced colon cancer cells. We found fewer cells stained with DAPI in the CoNiPr (x ≤ 0.10) NSFs-treated group compared to the control cells (Figure 9A,B). The control cells (Figure 9A) showed normal and healthy cells, whereas the CoNiPr (x ≤ 0.10) NSFs-treated cells showed nuclear disintegration and chromatic fragmentation, which are signs of apoptosis or programmed cell death.

Recent advancements in nanotechnology have led to the development of nanotherapeutics, which are promising tools for cancer treatment. In this research paper, we investigated the efficacy of NSFs for cancer treatment in a cell line model. NSFs have shown promising results as potential agents for cancer treatment due to their magnetic properties and biocompatibility [7,31]. In particular, their ability to be targeted and localized within the body through magnetic fields has made them a promising candidate for targeted drug delivery in cancer therapy. The interaction between nanoparticles and cell surfaces is stimulated by attachment of nanoparticles to the cellular membranes, later internalized through the process of endocytosis, and agglomerated within the digestive vacuoles. Hence, it is possible to achieve cytotoxicity at increased concentrations due to overload of particles inside the cells. Furthermore, nano-spinel ferrites have been shown to exhibit a high degree of heating when exposed to an alternating magnetic field, making them useful for the hyperthermia treatment of cancer cells [32,33,34,35]. In addition, the unique physiochemical properties of nano-spinel ferrites make them suitable for multimodal imaging and cancer diagnosis. Overall, the potential applications of nano-spinel ferrites in cancer treatment are vast and hold great promise for improving patient outcomes [36,37,38]. However, their application in fungal treatment has become a recent research focus. Therefore, this study explores the potential of NSFs as a novel antifungal agent against *C. albicans*.

Despite promising results, more research is needed to fully understand the potential risks and benefits of nanotherapeutics and to ensure their safety and effectiveness before they can be widely used in clinical practice. The significant biological exploitation of nanoparticles would be practically possible once the related toxicity is controlled by tailoring the techniques. The synthesized novel nanomaterials studied for biomedical utilization needs an elaborated evaluation of its biocompatibility. A study showed that hemolysis is a significant blood compatibility examination due to the direct attachment of the nanoparticles with thrombocytes via bloodstream injection. A condition, called hemolysis takes place once the membrane of RBC is damaged, that leads to the loss of hemoglobin through the distorted membrane. Such condition leads to numerous critical health issues like renal toxicity, hypertension, and anemia [19]. In addition, regulations and guidelines must be established to govern the development and use of nanotherapeutics, ensuring ethical and responsible practices. As nanotherapeutics continue to evolve and become more complex, it is crucial for the scientific community to collaborate with regulatory bodies and policymakers to establish a comprehensive framework for the safe and effective use of nanotherapeutics in healthcare. This will ensure patient safety and facilitate the translation of nanotherapeutics from bench to bedside, ultimately improving health outcomes and advancing modern medicine. Overall, nanotherapeutics have the potential to revolutionize healthcare and improve patient outcomes. Still, it is important to proceed cautiously and establish robust regulatory standards and guidelines to ensure the safe and ethical development, testing, and use of these innovative therapies.

Further research and development in this field are required to fully understand the potential of NSFs in biomedicine, including the optimization of their magnetic properties and formulation for clinical use.

## 3. Materials and Methods

### 3.1. Chemicals 

The hydrated nitrate salts of Fe(NO_3_)_3_·9H_2_O (≥98%, Merck, Darmstadt, Germany), Co(NO_3_)_2_·6H_2_O (≥98%, Merck, Darmstadt, Germany), Ni(NO_3_)_2_·6H_2_O (≥97%, Merck, Darmstadt, Germany), and PrCl_3_·xH_2_O (99%, Fischer, Espoo, Finland) were commercially obtained.

### 3.2. Synthesis and Characterization

For the synthesis of CoNiPr (x ≤ 0.10) NSFs, the ultrasonic approach was completed by dissolving the stoichiometric amounts of metal nitrates and chloride in 50 mL deionized (DI) water at 80 °C via continuous stirring for 1 h, and then its pH was adjusted to 11 using 2 M NaOH. The solution was put under ultrasonic irradiation with 20 kHz frequency and 70 W power using an ultrasonic homogenizer (UZ SONOPULS HD 2070) for 30 min. The final precursor was achieved by washing the powder several times with DI water and drying it out overnight at 90 °C [39]. No calcination was applied to the final products. A Rigaku Benchtop Miniflex (Cu K_α_) XRD (scanning 2θ range and step size were 20–70° and 0.0167°, respectively) was operated to obtain the powder patterns of products. Tescan Vega 3 (Tescan Orsay Holding, Brno, Czech Republic) scanning electron microscopes (SEM) were used for the surface analysis of the samples. The chemical composition of the samples was confirmed through EDX (energy dispersive X-ray) spectroscopy coupled with the SEM. The hydrodynamic particle size, polydispersity index, and zeta potential were measured using Malvern Zetasizer Pro (Malvern, UK).

### 3.3. Antifungal Assay

*Candida albicans* (American Type Culture Collection (ATCC) 14053) received from the Department of Microbiology, College of Medicine, Imam Abdulrahman Bin Faisal University, was considered to study the impact of CoNiPr (x ≤ 0.10) NSFs) on microbial cells. *C. albicans* was grown in Sabauraud broth (SDB) for 48 h at 37 ± 2 °C and harvested at exponential phase via centrifugation. Using sterile SDB, cells were adjusted to around 1 × 10^7^ CFU mL^−1^ for inoculum preparation to determine antifungal activity.

### 3.4. Colony-Forming Unit (CFU)

The antifungal activity was obtained by applying the CFU technique. The CoNiPr (x ≤ 0.10) NSFs) were sonicated and prepared using SDB culture medium to achieve a homogenized 16 mg/mL suspension. The samples were kept at 37 ± 2 °C for 48 h. *Candida*, incubated under the same parameters without the nanocomposite, was used as a control. Post incubation, the antifungal action was examined using the colony counting method. Precisely, the incubated *Candida* suspension was diluted to 1 × 10^4^ CFU/mL. In total, 100 µL of the diluted suspension was evenly spread on the SDA plates and incubated at 37 °C for 48 h. Post incubation, plates were observed and manually counted for the number of colonies and recorded to evaluate the viability of fungal cells treated with nanocomposites. The rate of the viability percentage (CV%) of the *Candida* was obtained using the given Equation (1):

Cell viability rate (%) = (A/B) × 100, Survival rate (%) = (A/B) × 100(1)

where A is the number of cells treated with nanomaterial and B is the number of untreated cells. 

### 3.5. Morphogenesis Studies Using SEM

A representative nanomaterial sample (x = 0.1) based on the cell viability studies was selected for the morphogenesis study. The candida cells were treated with CoNiPr (x ≤ 0.10) NSFs) and untreated cells were kept as the control, as described above. After the incubation of the test organism, the samples were fixed as per the protocol described by Rehman et al. [40].

### 3.6. In Vitro Evaluation of CoNiPr (x ≤ 0.10) NSFs)

Colorectal carcinoma HCT-116 and embryonic kidney cells HEK-293 were purchased from American Type Culture Collection (ATCC), Manassas, VA, USA. The cells were considered for anticancer activity after treatment with CoNiPr (x ≤ 0.10) NSFs) to examine cell viability. The cells (20,000/well) were first grown in a 96-well culture plate in the DMEM media comprised of fetal bovine serum + L-glutamine + penicillin + streptomycin + selenium in a CO_2_ incubator at 37 °C. Once the cells achieved 70–80% confluence, they were treated with CoNiPr (x ≤ 0.10) NSFs) with dosages (2.0 µg/mL to 500 µg/mL) for 48 h and were taken for the MTT assay [41]. The control group was not treated with CoNiPr (x ≤ 0.10) NSFs). Thereafter, cells were immersed in the MTT solution (5.0 mg/mL) and were subsequently treated with DMSO (1%). The optical density (OD) was read with an absorbance value of 570 nm on the microplate reader (Biotek Instruments) and % cell viability was calculated using Equation (2) [42]. The inhibitory concentration (IC50) was calculated using GraphPad Prism 8. The unit for the inhibitory concentration (IC_50_), expressed in µg/mL+, is the Standard Deviation. The statistical data presented were considered from triplicates, and the data were analyzed by using GraphPad Prism 10 Software, San Diego, CA, USA.

% cell viability = (Optical density (OD) of the sample)/Optical density (OD) of control) × 100(2)

### 3.7. Apoptotic Cell Death 

To examine the nuclei of the cancer cells post-treated with CoNiPr (x ≤ 0.10) NSFs at the concentration of IC_50_, cells at were kept in a CO_2_ incubator for 48 h and then treated with paraformaldehyde. The cells were then washed, stained with DAPI solution, and examined using a Fluorescence Confocal Scanning Microscope (Zeiss, Oberkochen, Germany) [43].

## 4. Conclusions

Magnetic nanoparticles have attracted immense interest within the biomedical field, including diagnosis and therapies. These nanoparticles are able to play a role in a drug delivery system by accumulating at the site via passive or active targeting. Passive targeting is mainly based on the permeability and retention (EPR) effect, while active targeting relies on the magnetic properties of nanoparticles through magnetic fields. Additionally, these nanoparticles have been applied as contrast materials in magnetic resonance imaging (MRI). Current in vitro studies have demonstrated that the synthesized Co_0.5_Ni_0.5_Pr_x_Fe_2−x_O_4_ (0.00 ≤ x ≤ 0.10) NSFs nanoparticles effectively inhibit the growth of colorectal cancer cells and *C. albicans*, suggesting their potential as a nanotherapeutic agent for treating colorectal cancer and candidaemia. Future studies should investigate the in vivo efficacy and toxicity of these nanoparticles, as well as their potential for targeted drug delivery. In conclusion, this study reinforces that synthesized Ni-Co nano-spinel ferrites doped with Pr^3+^ ions exhibit enormous potential as a biocompatible and effective nanotherapeutic agent for treating colorectal cancer and candidaemia. This study highlights the importance of nanotechnology in developing efficient and targeted therapies for various diseases, including cancer and fungal infections. Further research should focus on optimizing the synthesis process to enhance the efficacy of these nanoparticles and investigating their potential for clinical translation. Additionally, future studies should investigate the mechanism of action of these nanoparticles and their potential for combination therapy with existing anticancer and antifungal agents.

## Figures and Tables

**Table 1:** Textural and structural properties of CoNiPr (0.00 ≤ x ≤ 0.08) NSFs.

x	BET Surface Area (m^2^/g)	Pore Volume (cm^3^/g)	Average Pore Size (nm)
0.00	91	0.09	3.8
0.02	125	0.09	2.8
0.04	146	0.13	3.7
0.06	151	0.12	3.3
0.08	117	0.08	2.8

**Table 2:** Particle size and zeta potential of CoNiPr (x ≤ 0.00 and 0.08).

CoNiPr	Particle Size (nm)	Polydispersity Index	Zeta Potential (mV)
x = 0.00	1327	0.58	−6.13
x = 0.08	1923	0.47	−10.2

**Table 3:** Impact of CoNiPr (x ≤ 0.10) NSF treatment on HCT-16 cells (IC: inhibitory concentration).

Co_0.5_Ni_0.5_Pr_x_Fe_2-x_O_4_ (0.00 ≤ x ≤ 0.10) NSFs	HCT-11 (IC_50_) (µg/mL)
x = 0.00	63 ± 4.61
x = 0.02	227 ± 7.91
x = 0.04	57 ± 1.21
x = 0.06	106 ± 2.41
x = 0.08	46 ± 0.91
x = 0.10	288 ± 8.21

## Data Availability

Data is contained within the article.
