# Correction: Rehman et al. Pr^3+^ Ion-Substituted Ni-Co Nano-Spinel Ferrites: Their Synthesis, Characterization, and Biocompatibility for Colorectal Cancer and Candidaemia. *Pharmaceuticals* 2023, *16*, 1494

**DOI:** 10.3390/ph18040503

**Published:** 2025-03-31

**Authors:** Suriya Rehman, Balasamy Rabindran Jermy, Irfan A. Rather, Jamal S. M. Sabir, Suhailah S. Aljameel, Munirah A. Almessiere, Yassine Slimani, Firdos A. Khan, Abdulhadi Baykal

**Affiliations:** 1 Department of Epidemic Diseases Research, Institute for Research & Medical Consultations (IRMC), Imam Abdulrahman Bin Faisal University, Dammam 31441, Saudi Arabia; 2 Department of Nanomedicine Research, Institute for Research & Medical Consultations (IRMC), Imam Abdulrahman Bin Faisal University, Dammam 31441, Saudi Arabia; rjermy@iau.edu.sa; 3 Department of Biological Sciences, Faculty of Science, King Abdulaziz University, Jeddah 21589, Saudi Arabia; ammm@kau.edu.sa; 4 Center of Excellence in Bionanoscience Research, King Abdulaziz University, Jeddah 21589, Saudi Arabia; 5 Department of Chemistry, College of Science, Imam Abdulrahman Bin Faisal University, Dammam 31441, Saudi Arabia; ssaljameel@iau.edu.sa; 6 Department of Biophysics Research, Institute for Research & Medical Consultations (IRMC), Imam Abdulrahman Bin Faisal University, Dammam 31441, Saudi Arabia; malmessiere@iau.edu.sa (M.A.A.); yaslimani@iau.edu.sa (Y.S.); 7 Department of Stem Cell Research, Institute for Research & Medical Consultations (IRMC), Imam Abdulrahman Bin Faisal University, Dammam 31441, Saudi Arabia; fakhan@iau.edu.sa; 8 Food Engineering Department, Faculty of Engineering, Istanbul Aydin University, Florya, Istanbul 34295, Turkey; abaykal@iau.edu.sa

## Error in Figure

In the original publication [1], there was a mistake in Figure 2 as published. There are two SEM images that were unintentionally duplicated multiple times during the process of copying and pasting of SEM files. The corrected Figure 2 appears below. The authors state that the scientific conclusions are unaffected. This correction was approved by the Academic Editor. The original publication has also been updated.

## Figures and Tables

**Figure 2:**
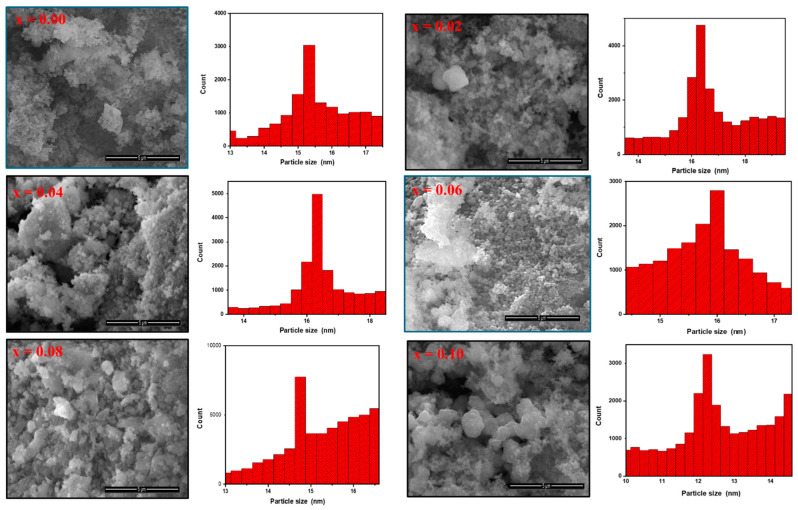
SEM surface analysis and grain size histograms of CoNiPr (0.00 ≤ x ≤ 0.10) NSFs.
